# Ultrathin amorphous carbon films synthesized by filtered cathodic vacuum arc used as protective overcoats of heat-assisted magnetic recording heads

**DOI:** 10.1038/s41598-018-27528-5

**Published:** 2018-06-25

**Authors:** J. Matlak, K. Komvopoulos

**Affiliations:** Department of Mechanical Engineering, University of California, Berkeley, California, 94720 USA

## Abstract

Despite numerous investigations of amorphous carbon (*a*-C) films, a comprehensive study of the feasibility and optimization of sub-5-nm-thick *a*-C films deposited onto the write pole of heat-assisted magnetic recording (HAMR) heads is lacking. The main objective of this study was to identify the role of pulse substrate bias voltage and C^+^ ion incidence angle on the structure and thickness of 1–4-nm-thick *a-*C films deposited by a rather new thin-film deposition method, known as filtered cathodic vacuum arc (FCVA). The cross-sectional structure of *a-*C films synthesized under various FCVA conditions was examined by high-resolution transmission electron microscopy (HRTEM), scanning transmission electron microscopy (STEM), and electron energy loss spectroscopy (EELS). It was found that film growth under process conditions of low-to-intermediate substrate bias voltage (in the range of −25 to −100  V), low ion incidence angle (10°), very short deposition time (6 s), and fixed other deposition parameters (65% duty cycle of substrate pulse biasing and 1.48 × 10^19^ ions/m^2^·s ion flux) yields *a-*C films of thickness ≤4 nm characterized by a significant content (~50–60 at%) of tetrahedral (*sp*^3^) carbon atom hybridization. A threshold where *sp*^3^ hybridization is greatly reduced due to limited film growth was determined from the HRTEM/STEM and EELS measurements. The results of this study demonstrate the viability of FCVA to produce extremely thin and uniform protective *a-*C films with relatively high *sp*^3^ contents for HAMR heads.

## Introduction

Ultrathin, amorphous carbon (*a*-C) films are extensively used as protective overcoats of magnetic recording media, because they demonstrate exceptional mechanical properties and structural stability^1^. Contemporary magnetic heads and hard disks are coated with a thin *a*-C film deposited by plasma-enhanced chemical vapor deposition^2,3^ or filtered cathodic vacuum arc (FCVA)^4–8^ to provide protection against damage and wear due to intermittent asperity contact^9^. The advent of new data storage technologies, such as heat-assisted magnetic recording (HAMR)^10,11^, motivated by increasing demands to boost the magnetic storage density beyond the 1 Tb/in^2^ barrier^12^, has brought new challenges in the development of suitable protective *a*-C films. The HAMR technology uses a laser-optical system integrated in the magnetic head and a near-field transducer (NFT) to focus a laser beam onto a small spot of the hard disk surface in order to locally heat the fine-grained high-magnetic-anisotropy medium above its Curie temperature^13^ and enable data to be stored by the write pole of the head in tightly packed magnetic nanodomains.

The absorption and dissipation of laser energy in the vicinities of the NFT and the write pole, however, raises the local temperature by several hundred degrees Celsius, increasing the propensity of mechanical wear and oxidation^14,15^ of the carbon film on the NFT and write pole. Additionally, thermal expansion of the NFT and write pole due to laser heating leads to the formation of a thermal protrusion^16^, which further reduces the flying height and increases the likelihood of contact at the head-disk interface. To compensate for the decrease of the head-disk physical spacing, further film thinning is necessary. However, because the protective film thickness is already approaching several atomic layers, preserving its continuity and desirable structural characteristics, such as high tetrahedral (*sp*^3^) atomic carbon hybridization, becomes challenging.

Most film deposition methods, including various physical vapor deposition and sputtering techniques, cannot produce continuous films of thickness equal to ~5 nm or less^17^. Film thinning is driven by the exponential dependence of storage density on the physical spacing between the magnetic medium of the hard disk and the write pole of the magnetic head^18^. FCVA is the only demonstrated and scalable low-temperature technique for synthesizing continuous and ultrathin *a*-C films with excellent nanomechanical/tribological properties, including high hardness and wear resistance^4^. Such films can withstand large temperature fluctuations while maintaining their structural stability^19,20^, mainly because of the dominance of *sp*^3^ hybridization. A significant fraction of *sp*^3^ carbon atom hybridization is critical for preventing rapid structural destabilization and graphitization of the film due to laser heating during the write process of a HAMR head.

The mechanical integrity of *a*-C films greatly depends on the fractions of trigonal (*sp*^2^) and *sp*^3^ atomic carbon hybridization, with harder films generally characterized by a higher *sp*^3^ content^1^. The dominance of specific C-C hybridization strongly depends on the C^+^ ion energy. The intense collisions between bombarding C^+^ ions and C atoms of the growing film encountered under deposition conditions of optimum ion energy (typically ~120 eV)^1,21^ promote *sp*^3^ hybridization without inducing significant thermal spikes that could result in *sp*^3^ → *sp*^2^ rehybridization. Previous studies of ultrathin *a*-C films synthesized by the FCVA method have shown the formation of a multilayered film structure comprising an interface (intermixing) layer, a buffer layer with gradually increasing *sp*^3^ fraction, a bulk layer of constant and high *sp*^3^ content, and a surface layer with *sp*^3^ hybridization sharply decreasing toward the film surface^22^.

Deposition of sub-5-nm-thick *a*-C films under optimal FCVA working conditions, however, is challenging because the thickness of the highly *sp*^3^ hybridized bulk layer, which controls the structure, thermal stability, nanomechanical/tribological properties, and oxidation resistance of the film, becomes comparable with the thicknesses of the *sp*^2^-rich intermixing and surface layers. This explains the increase of the *sp*^3^ content with the carbon film thickness^23^ and the use of seed underlayers to enhance *sp*^3^ hybridization and improve the tribological properties of *a*-C films^24,25^. The principal objective of this study, therefore, was to examine the growth of ≤4-nm-thick *a*-C films under FCVA conditions conducive to the formation of a sufficiently thick bulk layer with high *sp*^3^ content. While earlier studies focused on the effects of the duty cycle of substrate biasing^26^ and the ion incidence angle^27^ on the structure and growth of *a*-C films for the hard disk of HAMR drives, the present study is the first in a series of FCVA investigations to explore the structure and thickness of much thinner (≤4 nm) *a*-C films for the protection of the write pole of HAMR heads. In fact, this is the first comprehensive study to investigate FCVA deposition of *a*-C films on a FeNi substrate, the growth and structure of 1–4-nm-thick *a*-C films, and the simultaneous effects of ion incidence angle and substrate bias voltage on the structure of ultrathin *a*-C films. By selecting optimum values for some important deposition parameters (i.e., duty cycle of substrate pulse biasing and deposition time), which have been shown to enhance ion sputtering, subplantation^28^, and film thinning, parametric studies were carried out to identify the role of substrate bias voltage and C^+^ ion incidence angle on the multilayered structure, thickness, and *sp*^3^ content of ≤4-nm-thick *a*-C films for HAMR heads. This was achieved by performing detailed investigations of the film cross-sectional structure by high-resolution transmission electron microscopy (HRTEM), scanning transmission electron microscopy (STEM), and electron energy loss spectroscopy (EELS), and surface topography imaging by atomic force microscopy (AFM). Select HRTEM and STEM/EELS results are contrasted to elucidate the effects of primary FCVA deposition parameters on the structure and thickness of ultrathin *a*-C films deposited on FeNi substrates, resembling write pole stacking in HAMR heads.

## Results

### Film uniformity

**Figure 1:**
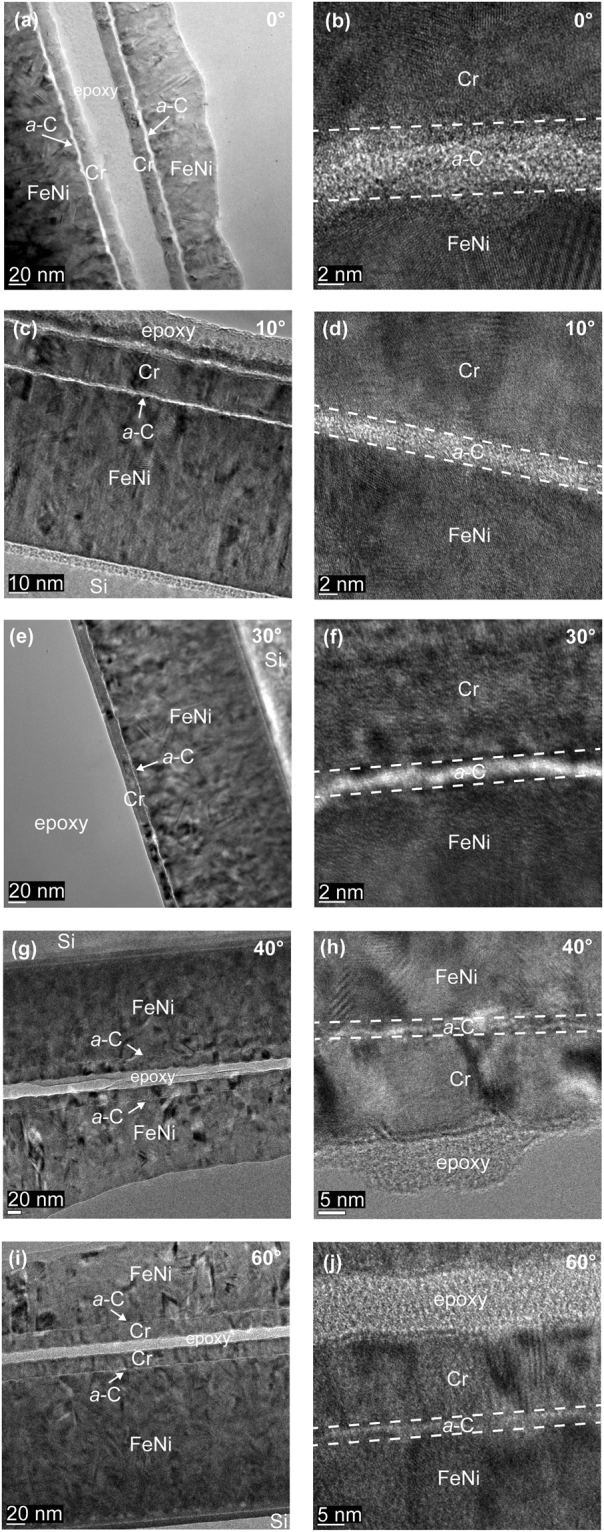
Cross-sectional HRTEM images of *a*-C films deposited under FCVA conditions of 65% duty cycle of substrate pulse biasing, −100 V substrate bias voltage, 6 s deposition time, and ion incidence angle between 0° and 60°.

Figure 1 shows HRTEM images of the cross-sectional structure of *a*-C films deposited under FCVA conditions of 65% duty cycle of substrate pulse biasing, −100 V substrate bias voltage, 6 s deposition time, and ion incidence angle between 0° and 60°. The low- and high-magnification images, shown on the left and right columns of Fig. 1, respectively, illustrate the order of sample stacking and the uniformity of the *a*-C films. Contrast differences reveal a layered cross-sectional structure consisting of crystalline Si substrate, FeNi base layer, *a*-C film, Cr capping layer, and epoxy bond layer. All films are shown to be conformal to the surface topography of the FeNi base layer. A comparison of the HRTEM images shown in the right column of Fig. 1 indicates a decrease in film thickness with increasing ion incidence angle, consistent with the results of a previous FCVA study of relatively thicker *a*-C films^27^. This trend can be attributed to the decrease of the penetration range of energetic C^+^ ions with increasing ion incidence angle. In fact, for a large ion incidence angle (i.e., ≥40°) the *a*-C film is barely visible even at a high magnification (Fig. 1h and j).

**Figure 2:**
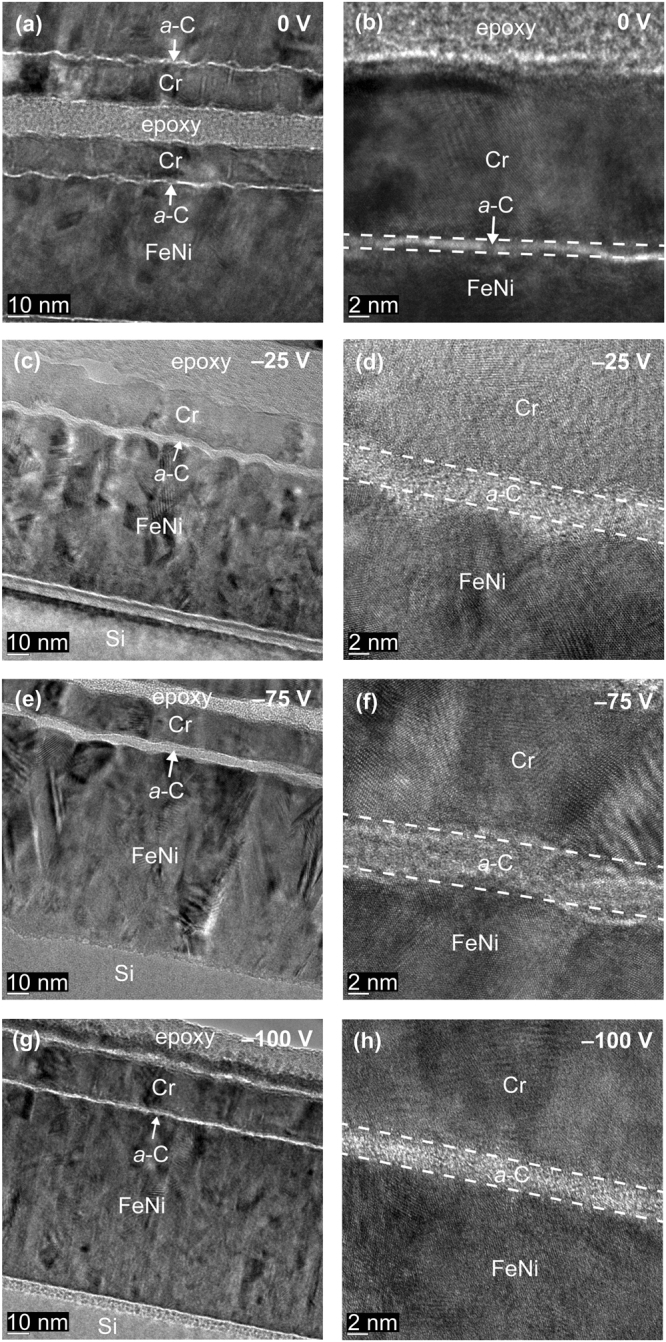
Cross-sectional HRTEM images of *a*-C films deposited under FCVA conditions of 65% duty cycle of substrate pulse biasing, 10° ion incidence angle, 6 s deposition time, and substrate bias voltage between 0 and −100 V.

**Figure 3:**
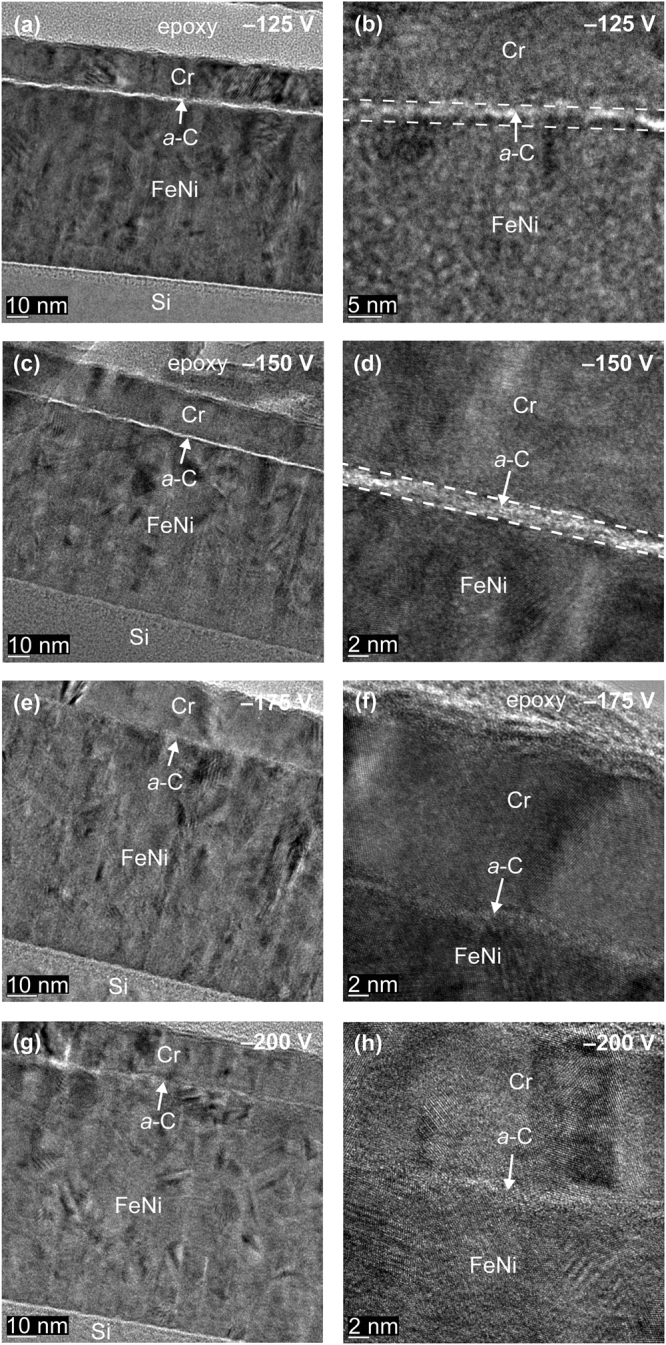
Cross-sectional HRTEM images of *a*-C films deposited under FCVA conditions of 65% duty cycle of substrate pulse biasing, 10° ion incidence angle, 6 s deposition time, and substrate bias voltage between −125 and −200 V.

Figures 2 and 3 show the effect of substrate bias voltage on the conformity and thickness of *a*-C films deposited under FCVA conditions of 65% duty cycle of substrate pulse biasing, 10° ion incidence angle, 6 s deposition time, and substrate bias voltage in the range 0 to −100 V and −125 to −200 V, respectively. Again, the low- and high-magnification images, shown in the left and right columns of Figs 2 and 3, respectively, confirm the continuity and conformability of the *a*-C films deposited onto the FeNi base layer. A comparison of the high-magnification HRTEM images shown in Figs 2 and 3 reveals an increase in film thickness with substrate bias voltage changing from 0 to −75 V, followed by a decrease in film thickness with substrate bias voltage varied from −75 to −200 V. These two opposite trends are attributed to the effect of kinetic energy of energetic C^+^ ions on film growth^29^.

### Film structure and thickness

**Figure 4:**
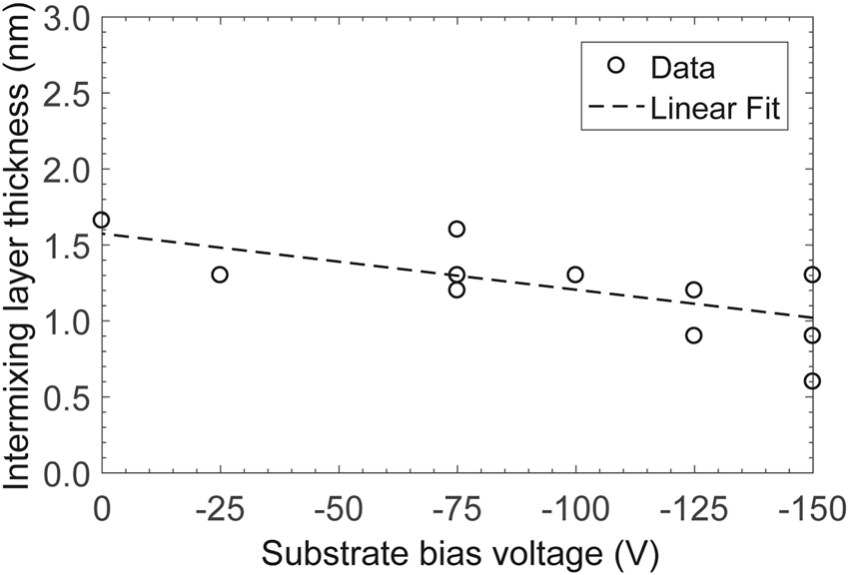
Thickness of intermixing layer versus substrate bias voltage for *a*-C films deposited under FCVA conditions of 65% duty cycle of substrate pulse biasing, 10° ion incidence angle, and 6 s deposition time.

The cross-section elemental composition and structure of the *a*-C films were obtained from STEM/EELS measurements. EELS uses the inelastic electron-electron collisions between electron beam electrons and inner (core-shell) sample electrons producing ionization edges to detect the presence of different elements. The C ionization edge is at 284 eV and reveals both *sp*^2^ and *sp*^3^ hybridization modes, which are identified by π* and σ* peaks in the energy range of 285–305 eV. The Fe and Ni ionization edges are in the energy range of 708–855 eV. Intermixing between carbon and substrate elements can be determined by identifying the regions in the high loss energy range of EELS spectra where the Fe L-edge and the Ni L-edge coexist with the C K-edge. For *a*-C films deposited under FCVA conditions of 65% duty cycle of substrate pulse biasing, 10° ion incidence angle, and 6 s deposition time, Fig. 4 shows intermixing of FeNi and C in a distance range of 0.5–2 nm, which is attributed to the subplantation process^28^. A linear fit to the data suggests a decreasing trend of intermixing layer thickness with the change in substrate bias voltage from 0 to −150 V. This is either due to the increasing contribution of resputtering relative to the knock-on mechanism that is dominant at higher C^+^ ion kinetic energies, or the increased dilution of carbon with increasing depth that cannot be resolved by EELS.

**Figure 5:**
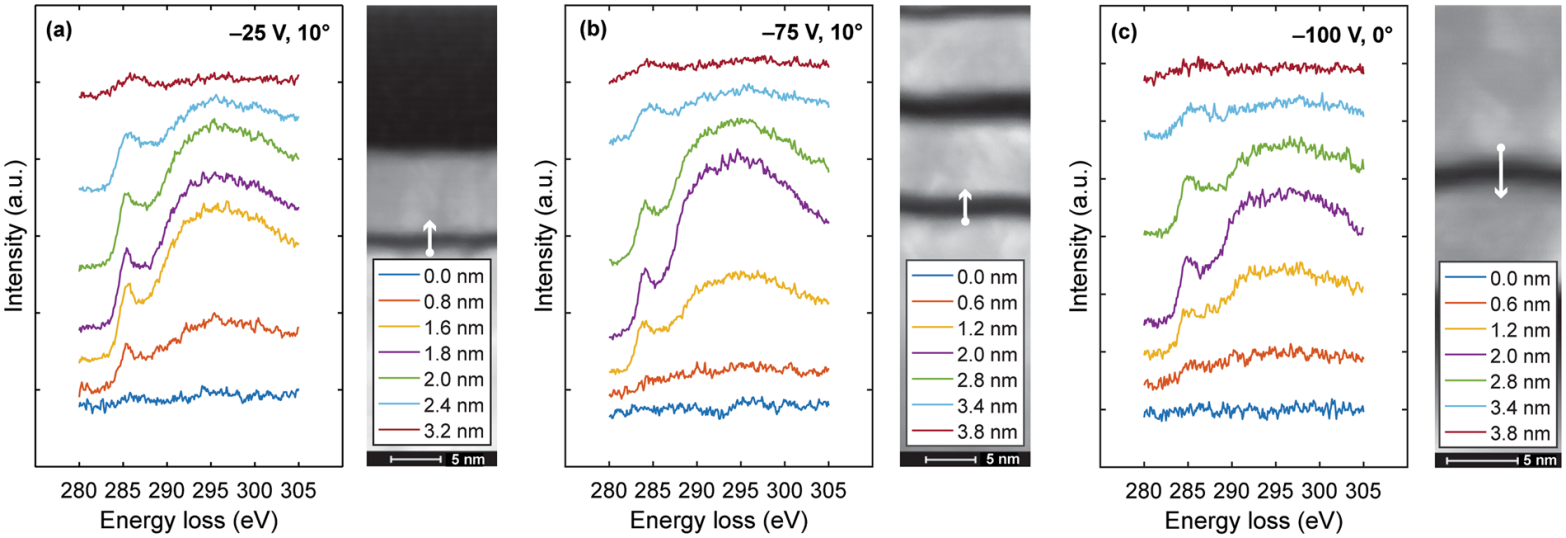
C K-edge EELS spectra of *a*-C films deposited under FCVA conditions of 65% duty cycle of substrate pulse biasing, 6 s deposition time, (**a**) −25 V substrate bias voltage and 10° ion incidence angle, (**b**) −75 V substrate bias voltage and 10° ion incidence angle, and (**c**) −100 V substrate bias voltage and 0° ion incidence angle.

Figure 5 shows high-energy EELS spectra of C K-edge obtained across the FeNi/*a*-C/Cr sample stack of *a*-C films exhibiting notable *sp*^3^ hybridization. These films were deposited under FCVA conditions of varying substrate bias voltage and ion incidence angle. The HRTEM images on the right side of Fig. 5 show the scanning direction from the FeNi base layer toward the Cr capping layer and the incremental distance between successive EELS spectra. Each spectrum was processed to remove background noise and calibrated by aligning the center of the π* peak at 285 eV. The spectra of the FeNi base layer and Cr capping layer do not contain neither π* or σ* peak, revealing the absence of carbon. In the intermixing region of FeNi with C, the increasing intensity of the π* and σ* peaks with decreasing distance from the *a*-C film reveals C subplantation in the FeNi base layer. Toward the center of the *a*-C film, the presence of a much larger σ* peak than π* peak indicates a greater fraction of *sp*^3^ hybridization in the bulk layer of the *a*-C film than the intermixing layer.

The *sp*^2^ and *sp*^3^ fractions can be calculated by measuring the π*/σ* peak area ratio of the sample ${({\pi }^{\ast }/{\sigma }^{\ast })}_{{\rm{sample}}}$ and a standard (std) sample ${({\pi }^{\ast }/{\sigma }^{\ast })}_{{\rm{std}}}$. Since 100 at% *sp*^2^ has one π* and three σ* orbitals, while 100 at% *sp*^3^ has only four σ* orbitals, the fraction of *sp*^2^ bonded carbons atoms *x* in the sample is given by

1 $$\frac{{({\pi }^{\ast }/{\sigma }^{\ast })}_{{\rm{sample}}}}{{({\pi }^{\ast }/{\sigma }^{\ast })}_{{\rm{std}}}}=\frac{3x}{4-x}$$

The ${\pi }^{\ast }$ peak was fitted with a Gaussian distribution, while the ${\sigma }^{\ast }$ peak was integrated between 290 and 350 eV. The ${({\pi }^{\ast }/{\sigma }^{\ast })}_{{\rm{std}}}$ peak area ratio of the standard sample (pure graphite with 100 at% *sp*^2^) was found to be equal to 0.2.

**Figure 6:**
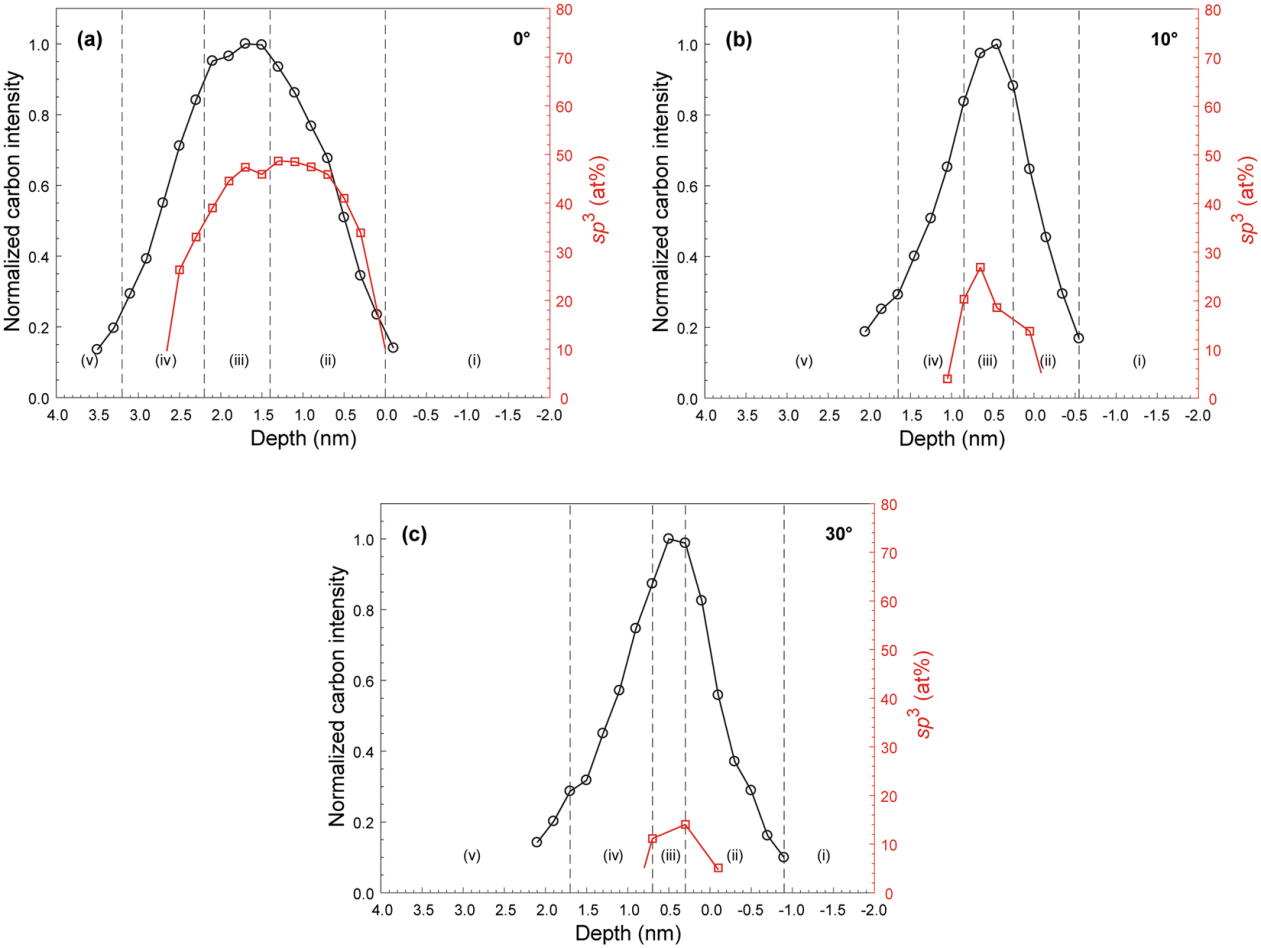
Depth profiles of normalized carbon intensity and *sp*^3^ fraction calculated from C K-edge EELS spectra of *a*-C films deposited under FCVA conditions of 65% duty cycle of substrate pulse biasing, −100 V substrate bias voltage, 6 s deposition time, and ion incidence angle equal to (**a**) 0°, (**b**) 10°, and (**c**) 30°. The dashed lines indicate the interfaces between adjacent layers comprising the multilayered cross-sectional structure of the *a*-C films.

**Figure 7:**
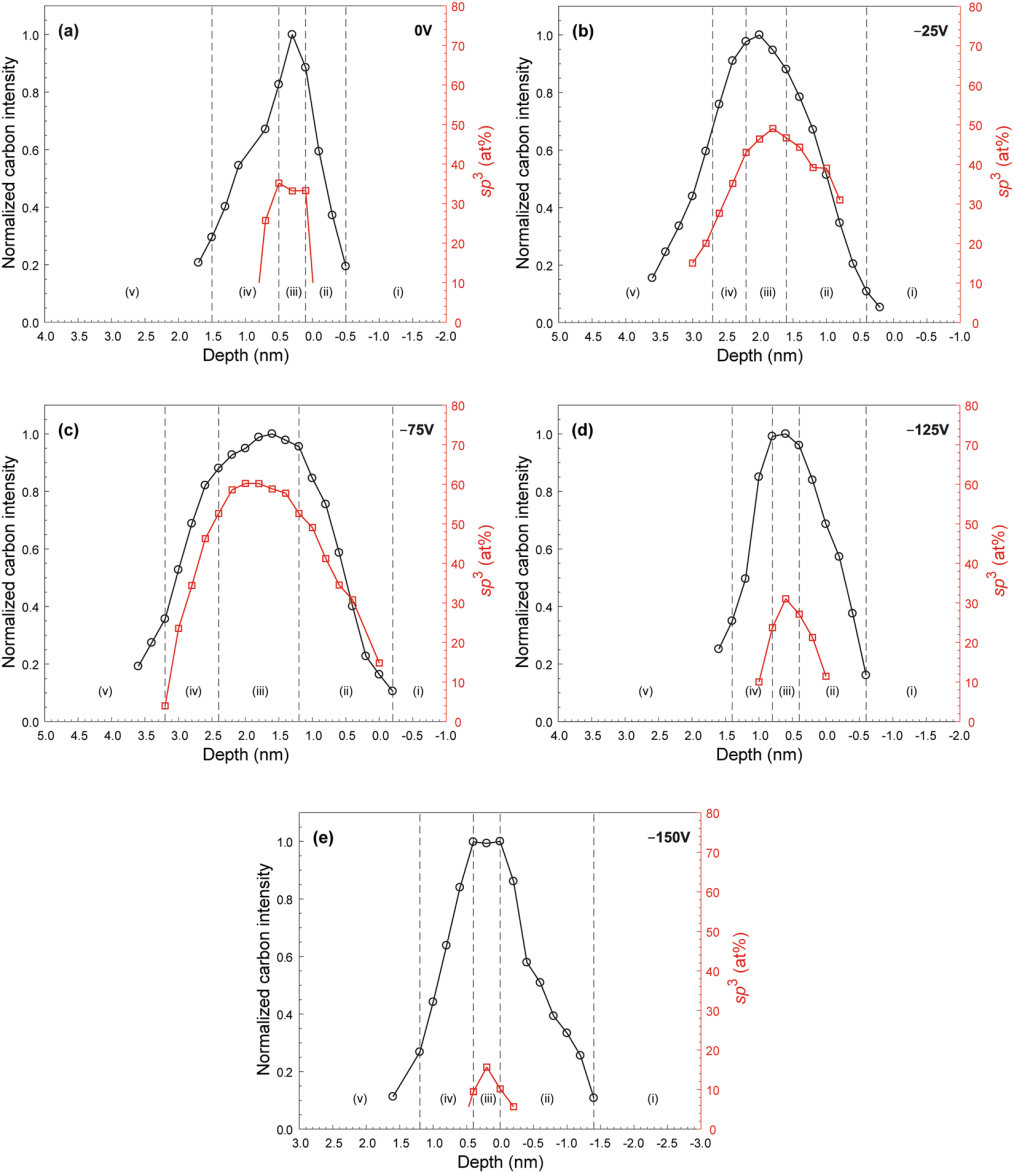
Depth profiles of normalized carbon intensity and *sp*^3^ fraction calculated from C K-edge EELS spectra of *a*-C films deposited under FCVA conditions of 65% duty cycle of substrate pulse biasing, 6 s deposition time, 10° ion incidence angle, and substrate bias voltage equal to (**a**) 0 V, (**b**) −25 V, (**c**) −75 V, (**d**) −125 V, and (**e**) −150 V. The dashed lines indicate the interfaces between adjacent layers comprising the multilayered cross-sectional structure of the *a*-C films.

Figures 6 and 7 show depth profiles of normalized C intensity and *sp*^3^ content of *a*-C films deposited under FCVA conditions of varying ion incidence angle and substrate bias voltage, respectively, 65% duty cycle of substrate pulse biasing, and 6 s deposition time. A multilayered cross-sectional film structure consisting of the following five layers can be seen in each sample:

(i) Base layer: The intensity of the C signal is approximately zero because it arises from the FeNi base layer.
(ii) Intermixing layer: The C concentration increases sharply showing a transition from low to modest values of *sp*^3^ hybridization.
(iii) Bulk layer: The C concentration stabilizes at ~100 at%, whereas the *sp*^3^ fraction remains fairly constant and at relatively high value.
(iv) Surface layer: The C concentration decreases sharply toward the film surface showing a transition from modest to low values of *sp*^3^ hybridization.
(v) Capping layer: The low intensity of the C signal is attributed to adventitious carbon.

Table 1 delineates the interfaces of above mentioned five layers in the deposited *a*-C films based on the normalized C-K edge intensity range, using the method introduced in a previous study^26^. Similar layers have been previously observed through the multilayered structure of thicker *a*-C films^22^.

**Table 1:** Layer interfaces in the multilayered structure of *a*-C films determined by the normalized C-K edge intensity or the *sp*^3^ content.

Interface	Range
Capping layer/surface layer	Normalized C-K edge intensity range: 0.3–0.4
Surface layer/bulk layer	Normalized C-K edge intensity range: 0.85–0.95
Bulk layer/intermixing layer	Normalized C-K edge intensity range: 0.9–1.0
Intermixing layer/substrate	*sp*^3^ <10 at% or normalized C-K edge intensity of <0.15

## Discussion

The multilayered *a*-C film structure revealed by HRTEM and STEM/EELS results can be interpreted in the context of the subplantation model^28^. The intermixing layer forms as a result of knock-on/recoil implantation and the penetration of the FeNi base layer by bombarding C^+^ ions. When a substrate bias voltage is applied, the energetic C^+^ ions impinging onto carbon deposited during the off-bias portion of the duty cycle knock C atoms to deeper locations and/or implant themselves into the FeNi base layer. Subsequent C^+^ ion impingement leads to the formation of a buffer layer on top of the intermixing layer where C^+^ ions are initially backscattered by atoms in the intermixing layer. This process continues until sufficient film growth decreases the likelihood of C^+^ ion and FeNi atom interaction, increasing the probability of only C-C atom interaction that is conducive to *sp*^3^ hybridization. (Because of the very small film thickness it is hard to distinguish the buffer layer in the EELS spectra; thus, this layer is considered together with the intermixing layer as one layer). The bulk layer is a result of further C^+^ ion subplantation where only C-C atom interaction is encountered. Subsequent C^+^ ion impingement onto the bulk layer generates a highly compressive stress environment, which promotes *sp*^3^ hybridization. The surface layer forms during the final stage of ion bombardment and is largely dominated by *sp*^2^ hybridization because of the lower C density and relatively less energetic C^+^ ion bombardment.

The film thickness and maximum *sp*^3^ content of the bulk layer (measured from HRTEM and STEM/EELS) versus deposition conditions are given in Table 2. The variation of the film thickness with substrate bias voltage and ion incidence angle for a short deposition time of 6 s can be explained by considering the effects of deposition, direct/recoil implantation, and sputtering. The kinetic energy of C^+^ ions increases with the magnitude of substrate bias voltage. Relatively low-magnitude substrate bias voltage produces less energetic C^+^ ions that favor film growth, whereas high-magnitude substrate bias voltage generates highly energetic C^+^ ions that enhance direct/recoil implantation and sputtering, which can be especially destructive at a very high C^+^ ion energy. The increase of the ion incidence angle leads to a lower compressive stress and less densification, in turn promoting *sp*^2^ hybridization. The overall decrease of the *sp*^3^ content of the bulk layer compared to thicker *a*-C films investigated in previous studies under similar deposition conditions^22,26,27^ may be attributed to the significantly shorter deposition time used in the present study. Because C^+^ ion bombardment produces a time dependent effect on the film structure, adequate ion bombardment is needed for *sp*^2^ → *sp*^3^ rehybridization or direct *sp*^3^ hybridization. The formation of an *sp*^3^-rich bulk layer is inhibited in the absence of sufficiently intense C-C atom interaction during ion bombardment. For the bulk layer to form, film growth must exceed the sum of intermixing and buffer layer thicknesses. Thus, without the formation of a sufficiently thick bulk layer with relatively high *sp*^3^ content, further film thinning cannot be achieved by simply decreasing the deposition time if the structural integrity of the *a*-C film is to be preserved. Therefore, to further decrease the film thickness, efforts must be directed toward minimizing the thickness of the *sp*^2^-rich intermixing and surface layers, while at the same time maintaining the dominance of the *sp*^3^-rich bulk layer. A possible method is to use a large ion incidence angle and/or low substrate bias voltage during initial deposition to decrease the intermixing layer thickness, switching later to a small ion incidence angle and/or high substrate bias voltage to generate deposition conditions conducive to *sp*^3^ hybridization. Decreasing the film thickness by a post-deposition treatment, such as Ar^+^ ion bombardment, is another possible method of film thinning^30^; however, it does not fully eliminate the surface layer because the top of the bombarded bulk layer undergoes *sp*^3^ → *sp*^2^ rehybridization upon sputter-etching of the surface layer.

**Table 2:** Effect of deposition parameters on the thickness and maximum *sp*^3^ content of the bulk layer of a-C films.

FCVA deposition conditions				HRTEM measurements	EELS measurements	
Deposition time	Duty cycle (%)	Incidence angle (deg.)	Substrate bias voltage (V)	Film thickness (nm)	Film thickness^*^ (nm)	Maximum *sp*^3^ content of bulk layer (at%)
6	65	0	−100	4.0 ± 0.3	3–4	49
		10	−100	2.3 ± 0.1	2–3	27
		30	−100	1.4 ± 0.1	2–3	14
		60	−100	1.1 ± 0.1	<1	<10
		10	0	1.0 ± 0.2	2–3	35
		10	−25	3.7 ± 0.2	3	49
		10	−75	5.0 ± 0.3	3–4	60
		10	−125	2.4 ± 0.2	2–3	31
		10	−150	1.8 ± 0.3	2–3	16
		10	−175	0.9 ± 0.2	<1	<10
		10	−200	—	<1	<10
12		10	−125	—	1–2	<10

^*^To account for noise and the presence of adventitious carbon, the formation of *a*-C film was determined when the normalized C-K edge intensity was above 0.1–0.2.

The present study is the first in a series of systematic FCVA investigations dealing with the effects of substrate pulse bias voltage and ion incidence angle on the cross-sectional structure and thickness of *a*-C films deposited on FeNi alloy, a common write pole material for HAMR heads. The parametric study of key deposition parameters performed in this investigation demonstrates the potential of FCVA to produce extremely thin *a*-C films that maintain the dominant effect of the *sp*^3^-rich bulk layer, which is critical to the mechanical integrity and structural stability of the *a*-C film, despite the significant decrease in film thickness. A comprehensive study of the thermal stability of the present *a*-C films will be presented in a forthcoming publication^31^.

## Conclusion

Ultrathin (1–4 nm) *a*-C films were deposited by FCVA under conditions of varying substrate bias voltage and C^+^ ion incidence angle. HRTEM and STEM/EELS results showed that *a*-C films of thickness ≤4 nm having a bulk layer with relatively high *sp*^3^ content (~50–60 at%) can be deposited under FCVA conditions of low to moderate substrate bias voltage (in the range of −25 to −125 V), small ion incidence angle (10°), and all other key deposition parameters set to optimum values. The obtained results indicate that film densification and growth of an *sp*^3^-rich bulk layer are critical factors in the formation of ultrathin *a*-C films characterized by relatively high (~60 at%) *sp*^3^ C-C atom hybridization. This is of paramount importance for the wear resistance and structural stability of ultrathin *a*-C films used as protective overcoats of HAMR heads.

## Methods

### Film deposition

A ~0.1-μm-thick base layer consisting of FeNi was sputtered onto commercial p-type Si(100) wafers in a low-pressure radio-frequency (RF) sputtering system (Randex-2400, Perkin-Elmer). The sputtering deposition process included the following three steps. First, the vacuum chamber was pumped down to a low pressure of ≤5 × 10^−6^ Torr to remove any residual gases adsorbed onto the chamber walls. Then, the FeNi alloy target and the Si(100) wafer were cleaned by Ar^+^ ion sputtering for 10 and 3 min, respectively, under conditions of 3 × 10^−3^ Torr working pressure, 250 W forward RF power, and 61 sccm Ar gas flow rate. Finally, a FeNi layer was sputtered onto the Si(100) wafer under plasma discharge conditions of 250 W adsorbed RF power, 0 substrate bias voltage, 3 × 10^−3^ Torr working pressure, 61 sccm Ar gas flow rate, and 5 min deposition time. The surface roughness of the FeNi base layer was measured from 2 × 2 μm^2^ images acquired with an AFM apparatus (Nanoscope II, Digital Instruments) operated in tapping mode, using a CoCr-coated tip with a 35 nm nominal radius of curvature. The root-mean-square roughness of the FeNi surface, averaged over at least five measurements obtained from different AFM surface scans, was found equal to 0.498 nm.

The FeNi-coated Si(100) wafers were cut into ~5 × 5 mm^2^ samples and cleaned by rinsing first in isopropanol for 10 min and then in acetone also for 10 min. Subsequently, the cleaned samples were coated with ultrathin *a*-C films in a custom-made FCVA system^4^. The FCVA deposition experiments comprised the following main steps. First, the vacuum chamber was pumped down to a low base pressure of <5 × 10^−7^ Torr to remove any residual gases adsorbed onto the chamber walls. Then, Ar gas was introduced into the chamber under a working pressure of 2 × 10^−4^ Torr, and the FeNi/Si(100) sample was subjected to a 2-min bombardment by 500-eV Ar^+^ ions (generated by a 64-mm Kauffman ion source) impinging onto the surface of the FeNi layer at an incidence angle of 60° (measured from the surface normal) to remove any surface contaminants and the native oxide layer. After this step, the base pressure was lowered to <5 × 10^‒7^ Torr and plasma arcing was induced at the cathode (99.99% pure graphite) surface by a mechanical striker. The plasma was stabilized by applying a cusp-configuration magnetic field to the cathode^4,5^. AFM imaging has shown that the magnetic field generated by the out-of-plane S-shaped electromagnetic coils of this system inhibits the deposition of macroparticles and/or droplets that may be ejected from the cathode surface during plasma arcing^32^. The current in the auxiliary, upstream, and downstream coils was set at ~32 A. Under these current plasma conditions, only high-purity (~99.99%) C^+^ ions arrive at the filter exit^5^. For one set of experiments, the ion incidence angle was varied between 0° and 60°, while all other conditions were set to optimal values, i.e., 1.48 × 10^19^ ions/m^2^·s ion flux, 6 s deposition time (8.88 × 10^19^ ions/m^2^ ion dose), −100 V pulsed substrate bias voltage, and 65% duty cycle of substrate pulse biasing, as determined from earlier studies^26^. For another set of experiments, the pulsed substrate bias voltage was varied between −25 and −150 V, while fixing the ion flux to 1.48 × 10^19^ ions/m^2^·s, the deposition time to 6 s (8.88 × 10^19^ ions/m^2^ ion dose), and the incidence angle to 10°. To enhance the film uniformity during deposition, the substrate holder was continuously rotated at an angular speed of 60 rev/min.

To distinguish the *a*-C film from the epoxy glue used to prepare the cross-sectional HRTEM/EELS samples, a 20-nm-thick Cr capping layer was deposited onto each carbon-coated sample. Sputtering of the Cr capping layer comprised the following steps. First, the vacuum chamber was pumped down to a low pressure of <5 × 10^−6^ Torr to remove any residual gases adsorbed onto the chamber walls. Then, Ar gas was introduced into the chamber, a working pressure of 3 × 10^−3^ Torr was established, and the Cr target was cleaned by Ar^+^ ion sputtering for 10 min under conditions of 3 × 10^−3^ Torr working pressure, 250 W forward RF power, and 61 sccm Ar gas flow rate. Finally, the Cr capping layer was sputter-deposited onto the surface of the carbon-coated samples by bombarding the Cr target with Ar^+^ ions for 1 min.

### Microanalysis methods

Cross-sectional HRTEM samples were prepared by mechanical grinding, dimpling, and surface finishing using ion milling. The samples were glued face-to-face with M-bond 610 epoxy and cured at 160 °C for 90 min. Additional Si(100) was epoxy-glued to both sides of the sandwiched samples to increase the thickness to >3 mm and cured also at 160 °C for 90 min. The samples were then sectioned with a diamond blade into approximately 0.7–1.0-mm-thick slices, and sawed and cored to 3-mm-diameter disks, which were polished down to a thickness of ~300 μm by successively using a smaller grit (30–0.1 μm) on a polishing system (MultiPrep, Allied High Tech). The center thickness of the disks was thinned down to <20 μm using a VCR Group Dimpler d500i. Finally, the disks were ion milled from the top and the bottom with Ar^+^ ion guns (PIPS II, Gatan) operated at 4.5 kV. An Ar^+^ ion incidence angle of 5° was used to produce a through-thickness hole across the sample/epoxy/sample interface stack.

HRTEM images and EELS spectra were obtained with a microscope (F20 UT, FEI Tecnai) operated at 200 kV, equipped with a CCD camera (2048 × 2048 pixels) that was positioned 42 mm behind a Gatan imaging filter. A 9.3-mrad C2 semi-angle and a 150-μm C2 aperture were used in this study. The EELS collection semi-angle was set at 16.3 mrad. Using the full width at half-maximum of the zero-loss peak, the energy resolution was found to range from 0.5 to 0.6 eV. Since the bandgap difference between *sp*^2^ and *sp*^3^ is ~0.8–0.9 eV, an energy resolution of 0.5–0.6 eV is sufficiently low to distinguish these hybridizations from each other. The spatial resolution in the STEM (without a monochromator) was 0.2 nm. For each sample, a scanning line was set perpendicular to the film interface, and data for each EELS spectrum were collected over an area of 0.2 nm diameter using a dwell time of 1 s. Each scan started in the FeNi base layer, preceded through the *a*-C film, and ended in the Cr capping layer.
